# Association of dietary inflammatory index with obesity among children and adolescents in the United States NHANES cohort: a population-based study

**DOI:** 10.1186/s13052-024-01586-0

**Published:** 2024-01-25

**Authors:** Lili Zhang, Huimin Peng, Yao Wang, Hongjun Ba

**Affiliations:** 1 Department of Pediatric Cardiology, Heart Centre, The First Affiliated Hospital, Sun Yat-sen University, Guangzhou, China; 2 Guangzhou Medical University Cancer Hospital, Guangzhou, China; 3 NHC key Laboratory of Assisted Circulation and Vascular Diseases, The First Affiliated Hospital, Sun Yat-sen University, Guangzhou, China

**Keywords:** Obesity, Dietary inflammatory index, Food, Adolescents, Children

## Abstract

**Background:**

Childhood obesity has become a huge challenge to childhood health, and there is a lack of understanding about the relationship between dietary inflammatory index (DII) and childhood obesity. The main objective of this study was to analyze the potential link between DII and obesity among children and adolescents residing in the United States.

**Methods:**

A cross-sectional analysis was performed using data obtained from the National Health and Nutrition Examination Survey between 2009 and 2018. In total, 12,454 participants were included in the analysis. DII was calculated based on dietary data from the first day of the 24-hour dietary recall. Logistic regression was used to analyze the association between DII and obesity, as well as central obesity defined by the waist-to-height ratio of 0.5 or higher or waist circumference ≥ 90th percentile for age and sex.

**Results:**

The mean dietary inflammation index was 2.05 (SE = 0.02), with higher levels in children than in adolescents (*P* = 0.01). According to our findings, the prevalence of central obesity was higher among adolescents (38.0%) than among children (31.4%). The adolescents in the third quartile of DII have a higher risk of overweight/obesity (OR = 1.46, 95% CI: 1.24–1.71) after adjusting for age, sex, and race. This positive association remained significant even after physical activity was added to the model. Concerning central obesity, the adolescents in the highest quartile of DII have a higher risk, independent of demographic characteristics and physical activity. However, no significant association was observed among children.

**Conclusions:**

The dietary inflammation index was positively associated with overweight/obesity and central obesity among adolescents in the United States after adjusting for confounding factors. These findings highlight the importance of promoting anti-inflammatory diets in adolescents to prevent obesity and its associated complications.

## Introduction

Childhood obesity is a major global, public health challenge, with implications for childhood hypertension, abnormal lipid metabolism, and abnormal glucose metabolism. It is also associated with the risk of developing chronic diseases such as coronary heart disease, cerebrovascular disease, and cancer in adulthood [1–4]. Although the mechanisms are not yet clear, there have been many valuable findings. In recent years, the concept of unhealthy obesity has been proposed in academia [5, 6]. It suggests that excessive accumulation of body fat, especially visceral fat, leads to adiposopathy, which is a significant cause of metabolic complications. Research has shown that the presence of adipose tissue dysfunction in obese individuals can lead to a state of inflammation in the body [7]. Studies indicate that inflammation may play a significant role in the development of metabolic disorders, cardiovascular diseases, and obesity-associated tumors [8, 9]. This is attributed to excess adipose tissue in obese individuals, which leads to increased production of cytokines and leptin while reducing anti-inflammatory immune cells [10, 11].

The Dietary Inflammation Index (DII) is a tool that assesses the inflammatory impact of a specific diet and has been associated with various chronic conditions [12–16]. This index incorporates a comprehensive assessment of the pro- and anti-inflammatory components of food, including macronutrients, minerals, vitamins, flavonoids, and specialty food constituents [13]. Currently, the DII is a standard tool used to assess diets internationally and across cultures.

Recently, there has been a surge in research on the correlation between DII and obesity. However, there is conflicting evidence regarding the findings of these studies. Certain studies have established a positive correlation between DII score and obesity in adults, indicating a higher likelihood of obesity with the consumption of pro-inflammatory foods. Nevertheless, other studies have yielded contradictory results [17, 18], while few studies have stated no significant relationships [19]. Limited studies conducted on DII and childhood obesity are often confined to a single age group and may not necessarily represent a larger population. Previous studies indicate conflicting results regarding the correlation between DII and obesity in children. A study conducted on 8–9 year–old children showed no significant association between DII, total body mass, and central obesity but showed a negative association with adipokine levels [20]. Another study of children aged 5–9 years discovered a positive association between DII and obesity [21]. In Iranian adolescents, a positive correlation between DII and body fat content has been reported [22], whereas a Brazilian study on 12–17-year-olds found an association between DII and overweight and abdominal obesity [23]. This is the largest study of its kind to date that analyzes data from a representative sample of American children and adolescents to investigate the potential link between DII and obesity.

## Methods

### Study design and Population

The analytical data utilized in this study were obtained from NHANES, specifically from the years 2009 to 2018. NHANES employs a complex, stratified, multistage probability sampling procedure to collect health and nutritional data. The data is gathered from a representative sample of the civilian, noninstitutionalized US population. The NHANES process involves a combination of a home interview and a physical examination, which includes a face-to-face 24-hour dietary recall interview conducted at a mobile examination center (MEC). Additionally, a second 24-hour dietary recall is administered via telephone 3 to 10 days later. The NHANES is an ongoing series of cross-sectional surveys that employ a multistage probability sampling method and oversample certain population segments [18]. Publicly available data from approximately 10,000 participants are released for each annual cycle [18]. Mobile clinics and home visits were used to conduct physical examinations, collect biological samples, and interview participants. The NCHS Research Ethics Review Committee approved the survey protocol and written informed consent was obtained from all participants. For detailed information on the NHANES design and procedures, please refer to previous studies [19, 24].

From 2009 to 2018, a total of 12,957 individuals participated in the MEC for the five-year cycles. Among them, 503 individuals did not complete the dietary survey, resulting in a total of 12,454 individuals included in the study. Age, gender, race, socioeconomic status, physical activity, energy intake, dietary components, and anthropometric measurements are some of the parameters that can be extracted from the NHANES database for the study participants.

### Adiposity measures

We used standard procedures and equipment to measure height, weight, and waist circumference [25]. BMI was calculated as weight in kilograms divided by height in meters squared. Further, We defined underweight, normal weight, overweight/obesity, obesity, and severe obesity according to age- and sex-specific percentile cutoffs acquired from CDC growth charts [26]. Underweight was defined as a BMI below the 15th percentile. Normal weight was defined as a BMI between the 15th and 85th percentile. Obesity was defined as BMI for age- and sex-specific categories at the 95th percentile or higher. Overweight/obesity was defined as a BMI at or above the age- and sex-specific 85th percentile of the 2000 CDC growth charts. Severe obesity was defined as a BMI at or above the age- and sex-specific 120% of the 95th percentile of the CDC growth charts published in 2000. According to NHANES III data, central obesity was defined as a waist circumference equal to or greater than the sex and age-specific 90th percentile, or a waist-to-height ratio of 0.5 or higher [27].

### Dietary inflammatory index

The development and validation of the DII have been thoroughly documented in other publication [28]. DII was calculated using dietary data collected during 24 h on day 1 [14, 29, 30]. This included information on the type and amount of food and drinks consumed during the preceding 24 h (midnight to midnight), which were collected by MEC. Subsequently, the data were used to approximate the intake of energy, nutrients, and food components from foods and beverages, as validated by the Working Group on Nutritional Methods [31, 32] and explained in another report [13].

The DII, which consists of 28 dietary components, was calculated based on the protocol provided by Shivappa et al. [28]. This index aims to evaluate the level of systemic inflammation by using six important inflammation markers (IL-1β, IL-6, IL-4, IL-10, TNF-α, and CRP). In this study, a score of “+1” was assigned if a dietary component increased the levels of CRP, TNF-α, IL-1β, and IL-6 or reduced the levels of IL-4 and IL-10. Conversely, a score of “-1” was assigned if a dietary component decreased the levels of CRP, TNF-α, IL-1β, and IL-6 or increased the levels of IL-4 and IL-10. Each individual food parameter was then multiplied by its respective effect score derived from the literature review. The sum of all food parameter-specific DII scores yielded the overall DII score for each participant, calculated as DII = b1 * n1 + b2 * n2…b28 * n28. Here, “b” refers to the literature-derived inflammatory effect score for each food parameter, and “n” refers to the food parameter-specific percentiles obtained from the dietary data derived from the FFQ (food frequency questionnaire). The pooled DII represents the pro-inflammatory or anti-inflammatory potential of an individual’s daily diet. Additionally, we employ imputation methods to infer missing food parameters [28].

To account for the total energy intake, the E-DII was calculated for every 1000 calories of food consumed, necessitating the use of an energy-standardized version of the global database.

### Covariates

The NHANES used several covariates to adjust for individual characteristics, including age, sex, race, ethnicity, BMI, physical activity, and poverty-income ratio. The CDC website provides detailed information on the measurement procedures. Physical activity data adhered to the World Health Organization guidelines [33]. According to the 2018 Physical Activity Guidelines Advisory Committee scientific report [34, 35], participants who reported less than 10 min of moderate-to-vigorous physical activity per week were labeled as inactive.

### Statistical analyses

The study conducted descriptive analyses on the entire sample and further stratified the data by age group, specifically children between 6 and 10 years old and adolescents between 11 and 19 years old. Results were presented as weighted means or percentages with standard errors. The main exposure variable was the estimated intake of DII. Multiple logistic regression was performed to investigate the relationship between DII and weight-related measures (overweight/obesity, obesity, and central obesity). Odds ratios were calculated and sorted into quartiles, allowing for comparisons across different DII percentiles. Combined dietary sample weights were used during statistical analyses performed using SAS version 9.4, or SAS-callable SUDAAN to account for non-response and sampling design. All statistical tests were conducted using a two-sided approach, and statistical significance was defined as *P* < 0.05.

## Results

Table 1 illustrates the sample size and baseline characteristics of the US children and adolescents who participated in the 2009–2018 NHANES. Children aged 6–11 represented 55.7% of the total sample, whereas adolescents aged 12–19 represented 44.3%. Interestingly, there was an almost equal distribution between the sexes, with 49.1% girls and 50.9% boys. Furthermore, no significant differences in race or poverty income ratios were observed between the children and adolescents (Table 1).

**Table 1:** Baseline characteristics of US children and adolescents aged 6 to 19 years, NHANES 2009-2018^a^

	All, 6-19 years		Aged 6–11 years		Aged 12–19 years		
	*n*	Mean or prevalence (SE)	*n*	Mean or prevalence (SE)	*n*	Mean or prevalence (SE)	*P* ^d^
Overall, n (%)	12,454	100%	6933	55.67%	5521	44.33%	
Age,y	12,454	12.48(0.06)	6933	9.03(0.03)	5521	15.90(0.04)	< 0.0001
Sex							0.96
Female	6118	48.96(0.02)	3427	48.92(0.90)	2691	48.99(0.89)	
Male	6336	51.04(0.02)	3506	51.08(0.90)	2830	51.01(0.89)	
Race/Ethnicity, %^b^							0.06
White (non-Hispanic)	3437	53.43(0.03)	1917	52.44(2.18)	1520	54.42(2.01)	
Non-Hispanic black	3038	14.02(0.01)	1681	13.75(1.05)	1357	14.28(1.16)	
Mexican-American	2751	15.29(0.01)	1546	15.75(1.44)	1205	14.84(1.31)	
Other Hispanic other	3228	17.26(0.01)	1789	18.06(0.90)	1439	16.46(0.76)	
Poverty income ratio, %^c^							0.38
< 130%	4993	30.10(0.01)	2833	33.16(1.39)	2160	31.87(1.59)	
> 130%	6343	62.46(0.03)	3528	66.84(1.39)	2815	68.13(1.59)	
Physical activity							< 0.0001
Active	4716	42.95(0.02)	4167	10.34(0.52)	549	75.29(1.02)	
Inactive	7738	57.05(0.02)	1354	89.66(0.52)	6384	24.71(1.02)	
Dii	12,454	2.05(0.02)	6933	2.11(0.03)	5521	1.99(0.03)	0.01
Energy intake, kcal	12,454	2042.72(11.35)	6933	1926.20(11.75)	5521	2148.05(18.50)	< 0.0001

^a^All estimates are weighted except sample sizes (n)

^b^Estimates from participants who reported other race-Hispanic origin or more than one race group are not presented separately

^c^Poverty income ratio was calculated as total family income relative to the Department of Health and Human Services poverty guidelines and was categorized as ≤ 130% or > 130%

^d^For categorical variables, we used Rao-Scott F-adjusted χ2 test. A t test was used to examine whether means of continuous variables varied by age groups. All tests were two-sided

Table 1 shows that the average DII was 2.05 (SE = 0.02), which was significantly higher in the children compared with adolescents (*P* = 0.01). Energy intake was significantly higher in adolescents than in children (*P* < 0.0001). Furthermore, Table 2 indicates that central obesity diagnosed using the WtHR was observed more frequently in adolescents than in children (38.0% vs. 31.4%). In contrast, there was no significant difference in the percentage of overweight/obesity between the two groups (40.7% vs. 40.3%). Additionally, the prevalence of severe obesity was slightly higher in adolescents (9.5%) than in children (8.3%) (Table 2).

**Table 2:** Anthropometric measurements among US children and adolescents, NHANES 2009-2018^a^

	All, 6-19years		Aged 6–11 years		Aged 12–19 years		
	n	Mean or prevalence(SE)	n	Mean or prevalence(SE)	n	Mean or prevalence(SE)	*P* ^f^
WtHR	12,454	0.49(0.00)	6933	0.48(0.00)	5521	0.50(0.00)	< 0.0001
Weight category							0.04
Underweight	311	2.79(0.00)	163	2.76(0.26)	148	2.87(0.37)	
Normal weight	6977	57.68(0.02)	3907	58.64(0.88)	3070	57.80(0.87)	
Overweight	2173	17.30(0.01)	1197	17.26(0.56)	976	17.65(0.63)	
Obesity	1772	13.57(0.00)	1048	14.43(0.48)	724	12.95(0.67)	
Severe obesity^b^	1101	7.74(0.00)	574	6.90(0.46)	527	8.73(0.53)	
Overweight/obesity^c^							0.67
Yes	5046	38.60(0.01)	2819	38.35(0.89)	2227	38.85(0.89)	
No	7408	61.40(0.02)	4114	61.65(0.89)	3294	61.15(0.89)	
Central obesity^d^							< 0.0001
Yes	4277	33.03(0.01)	2180	30.94(0.92)	2097	37.83(0.95)	
No	7663	62.98(0.02)	4468	69.06(0.92)	3195	62.17(0.95)	
Central obesity^e^							0.003
Yes	2473	18.68(0.01)	1500	20.19(0.72)	973	17.18(0.82)	
No	9981	81.32(0.03)	5433	79.81(0.72)	4548	82.82(0.82)	

^a^Data are reported as weighted mean (SE) for continuous variables or numbers and weighted percentage (SE for categorical variables

^b^Severe obesity was defined as a BMI at or above the age- and sex-specific 120% of the 95th percentile of the CDC growth charts published in 2000. Of the 1772 with obesity, 1101 had severe obesity

^c^Overweight/obesity was defined as a BMI at or above the age- and sex-specific 85th percentile of the 2000 CDC growth charts

^d^Central obesity was defined as WtHR ≥ 0.5.

^e^Central obesity was defined as waist circumferences of the 90th percentile or greater for the same age and sex based on the data from NHANES III (1988–1994)

^f^For categorical variables, we used Rao-Scott F-adjusted χ2 test. A t test was used to examine whether mean of WtHR varied by age groups. All tests were two-sided

WtHR, waist to height ratio

Table 3 reveals the correlation between estimated weight gain and overweight/obesity in children and adolescents. Among adolescents, the adjusted odds ratio of overweight/obesity in DII quartile three was 1.46 (95% CI: 1.24–1.71) compared to the lowest quartile, and the adjusted OR values remained significant (1.42 (95% CI: 1.21–1.67)(*P* < 0.0001)) after adjusting for age, sex, and race (Table 3, Model 2). The positive correlation remained significant when physical activity was added to the model to adjust for potential false positives (Table 3, Model 3). However, no significant association was observed in overweight or obese children (Table 3).

**Table 3:** Adjusted odds ratio (95% CI) of overweight/obesity in US children and adolescents NHANES 2009–2018

Midvalue of quartiles of estimated DII					
	Q1 OR (95% CI)	Q2 OR (95% CI)	Q3 OR (95% CI)	Q4 OR (95% CI)	*p* ^*a*^
*Aged 6–11 years* ^*b*^	1,372	1,575	1,543	1,448	
Model 1	Ref	1.067(0.921–1.236)	0.898(0.774–1.042)	0.871(0.748–1.014)	0.013
Model 2	Ref	1.093(0.942–1.267)	0.917(0.789–1.065)	0.884(0.758–1.030)	0.021
Model 3	Ref	1.092(0.942–1.267)	0.918(0.790–1.067)	0.887(0.761–1.034)	0.024
*Aged 12–19 years* ^*b*^	1,206	1,240	1,337	1,206	
Model 1	Ref	1.225(1.044–1.437)	1.464(1.250–1.716)	1.420(1.214–1.662)	< 0.0001
Model 2	Ref	1.221(1.040–1.434)	1.461(1.245–1.714)	1.427(1.217–1.673)	< 0.0001
Model 3	Ref	1.218(1.037–1.431)	1.456(1.241–1.708)	1.412(1.204–1.656)	< 0.0001

Overweight/obesity was defined as a BMI at or above the 85th percentile of the same age and sex based on the 2000 CDC growth charts. Model 1, adjusted for age, sex; model 2, adjusted for age, sex, race; model 3, adjusted for covariates of model 2 plus physical activity. ^a^*P* value for trend across percentiles of estimated usual sodium intake based on Satterthwaite adjusted-F test; all tests were two-sided. ^b^This row contains midvalue of quartiles of estimated dii. OR, odds ratio. Dii, dietary inflammatory index

Table 4 discloses that after adjusting for various confounding variables, the positive correlation between DII and obesity among adolescents was more pronounced and increased with increasing DII quartiles compared with overweight or obesity in the same age group. Conversely, no such correlation was observed among children after making the same adjustments.

**Table 4:** Adjusted odds ratio (95% CI) of obesity in US children and adolescents NHANES 2009–2018

Midvalue of quartiles of estimated DII					
	Q1 OR (95% CI)	Q2 OR (95% CI)	Q3 OR (95% CI)	Q4 OR (95% CI)	*p* ^*a*^
*Aged 6–11 years* ^*b*^	1,372	1,575	1,543	1,448	
Model 1	Ref	1.07(0.903–1.269)	0.959(0.807–1.140)	0.959(0.805–1.144)	0.38
Model 2	Ref	1.091(0.919–1.294)	0.977(0.821–1.163)	0.971(0.813–1.159)	0.451
Model 3	Ref	1.091(0.919–1.294)	0.978(0.822–1.164)	0.975(0.816–1.163)	0.48
*Aged 12–19 years* ^*b*^	1,415	1,206	1,240	1,337	
Model 1	Ref	1.299(1.073–1.572)	1.466(1.214–1.770)	1.564(1.299–1.883)	< 0.0001
Model 2	Ref	1.286(1.061–1.558)	1.444(1.194–1.745)	1.543(1.279–1.862)	< 0.0001
Model 3	Ref	1.28(1.057–1.551)	1.435(1.187–1.735)	1.515(1.255–1.829)	< 0.0001

Obesity was defined as a BMI at or above the 95th percentile of the same age and sex based on the CDC growth charts published in 2000

Model 1, adjusted for age, sex; model 2, adjusted for age, sex, race; model 3, adjusted for covariates of model 2 plus physical activity. ^a^*P* value for trend across percentiles of estimated usual sodium intake based on Satterthwaite adjusted-F test; all tests were two-sided. ^b^This row contains midvalue of quartiles of estimated dii. OR, odds ratio. Dii, dietary inflammatory index

In adolescents, DII had a positive correlation with central obesity, as measured by WC. Those in the highest quartile of DII had an increased likelihood of central obesity compared with those in the lowest quartile, even after adjusting for demographic characteristics and physical activity (adjusted OR = 1.56, 95%CI:1.27–1.93, *P* < 0.0001), as shown in Table 5, Model 3. This correlation remained significant when central obesity was defined as WtHR ≥ 0.5 (adjusted OR = 1.52, 95%CI:1.29–1.80, *P* < 0.0001), as displayed in Table 6, Model 3. However, we did not observe any noteworthy associations among children. In addition, there was no sex difference in the relationship between DII and obesity in children or adolescents (Table 7).

**Table 5:** Adjusted odds ratio (95% CI) of central obesity(WC ≥ 90th percentile) in US children and adolescents NHANES 2009–2018

Midvalue of quartiles of estimated DII					
	Q1 OR (95% CI)	Q2 OR (95% CI)	Q3 OR (95% CI)	Q4 OR (95% CI)	*p* ^*a*^
*Aged 6–11 years* ^*b*^	1,372	1,575	1,543	1,448	
Model 1	Ref	1.079(0.906–1.285)	0.955(0.800-1.141)	0.962(0.803–1.152)	0.381
Model 2	Ref	1.111(0.932–1.324)	0.981(0.820–1.173)	0.983(0.820–1.178)	0.503
Model 3	Ref	1.111(0.932–1.324)	0.983(0.822–1.175)	0.987(0.824–1.184)	0.541
*Aged 12–19 years* ^*b*^	1,206	1,240	1,337	1,206	
Model 1	Ref	1.279(1.032–1.585)	1.571(1.276–1.935)	1.614(1.313–1.983)	< 0.0001
Model 2	Ref	1.265(1.020–1.570)	1.559(1.265–1.923)	1.603(1.302–1.974)	< 0.0001
Model 3	Ref	1.257(1.013–1.560)	1.547(1.254–1.909)	1.569(1.273–1.934)	< 0.0001

Central obesity was defined as WC ≥ 90th percentile for the same age and sex

Model 1, adjusted for age, sex; model 2, adjusted for age, sex, race; model 3, adjusted for covariates of model 2 plus physical activity. ^a^*P* value for trend across percentiles of estimated usual sodium intake based on Satterthwaite adjusted-F test; all tests were two-sided. ^b^This row contains midvalue of quartiles of estimated dii. OR, odds ratio. WtHR, waist to height ratio. Dii, dietary inflammatory index

**Table 6:** Adjusted odds ratio (95% CI) of central obesity(WtHR ≥ 0.5) in US children and adolescents NHANES 2009–2018

Midvalue of quartiles of estimated DII					
	Q1 OR (95% CI)	Q2 OR (95% CI)	Q3 OR (95% CI)	Q4 OR (95% CI)	*p* ^*a*^
*Aged 6–11 years* ^*b*^	1,372	1,575	1,543	1,448	
Model 1	Ref	1.228(1.049–1.437)	1.062(0.905–1.245)	1.039(0.883–1.222)	0.801
Model 2	Ref	1.294(1.103–1.518)	1.11(0.944–1.305)	1.08(0.916–1.273)	0.889
Model 3	Ref	1.293(1.102–1.517)	1.113(0.947–1.309)	1.086(0.921–1.281)	0.825
*Aged 12- 19years* ^*b*^	1,206	1,240	1,337	1,206	
Model 1	Ref	1.243(1.054–1.466)	1.435(1.219–1.691)	1.456(1.238–1.712)	< 0.0001
Model 2	Ref	1.273(1.077–1.504)	1.497(1.267–1.768)	1.558(1.321–1.839)	< 0.0001
Model 3	Ref	1.266(1.071–1.497)	1.486(1.258–1.756)	1.528(1.294–1.803)	< 0.0001

Central obesity was defined as WtHR ≥ 0.5.

Model 1, adjusted for age, sex; model 2, adjusted for age, sex, race; model 3, adjusted for covariates of model 2 plus physical activity. ^a^*P* value for trend across percentiles of estimated usual sodium intake based on Satterthwaite adjusted-F test; all tests were two-sided. ^b^This row contains midvalue of quartiles of estimated dii. OR, odds ratio. Dii, dietary inflammatory index

**Table 7:** Adjusted odds ratio (95% CI) of obesity in US children and adolescents by Sex NHANES 2009–2018

	Midvalue of quartiles of estimated DII				
	Q1 OR (95% CI)	Q2 OR (95% CI)	Q3 OR (95% CI)	Q4 OR (95% CI)	p^a^
Aged 6–11 *years*^*b*^ male					
Model 1	Ref	1.25(0.991,1.576)	1.115(0.879,1.414)	0.929(0.720,1.198)	0.491
Model 2	Ref	1.25(0.990,1.576)	1.115(0.878,1.414)	0.938(0.727,1.211)	0.538
Model 3	Ref	1.276(1.000,1.628)	1.106(0.861,1.420)	0.953(0.729,1.246)	0.565
Aged 6–11 *years*^*b*^ female					
Model 1	Ref	0.928(0.720,1.196)	0.838(0.649,1.082)	0.969(0.755,1.244)	0.707
Model 2	Ref	0.928(0.721,1.196)	0.836(0.647,1.080)	0.967(0.753,1.241)	0.69
Model 3	Ref	0.859(0.660,1.118)	0.798(0.612,1.041)	0.907(0.699,1.177)	0.465
Aged12-19 *years*^*b*^ male					
Model 1	Ref	1.342(1.052,1.713)	1.646(1.285,2.108)	1.495(1.155,1.936)	< 0.001
Model 2	Ref	1.337(1.047,1.706)	1.639(1.279,2.099)	1.475(1.139,1.910)	< 0.001
Model 3	Ref	1.296(1.004,1.672)	1.676(1.293,2.174)	1.473(1.122,1.935)	< 0.001
Aged 12–19 *years*^*b*^ female					
Model 1	Ref	1.201(0.876,1.646)	1.27(0.938,1.719)	1.525(1.142,2.037)	0.003
Model 2	Ref	1.195(0.871,1.639)	1.259(0.929,1.705)	1.49(1.115,1.992)	0.006
Model 3	Ref	1.26(0.899,1.767)	1.372(0.995,1.892)	1.537(1.127,2.097)	0.006

Model 1, adjusted for age, race; model 2, adjusted for age, race and physical activity; model 3, adjusted for covariates of model 2 plus physical activity. ^a^*P* value for trend across percentiles of estimated usual sodium intake based on Satterthwaite adjusted-F test; all tests were two-sided. ^b^This row contains midvalue of quartiles of estimated dii. OR, odds ratio. Dii, dietary inflammatory index

## Discussion

Our study utilized NHANES 2009–2018 data and revealed that a higher DII was linked to increased odds of being overweight and/or obese. In addition, we observed a positive correlation between the DII score and central obesity in adolescents, even after adjusting for age, sex, and race. However, we did not find a significant association between DII and obesity in children in the US. Our findings are consistent with those of previous studies on childhood and adolescent obesity [20, 21, 36]. Although the exact reasons for the difference in the association between DII and obesity in children and adolescents remain uncertain, factors such as changes in hormone levels during puberty and the duration of obesity may play a significant role [37, 38]. Furthermore, the specific reasons for the association between the third quartile of DII and higher odds ratio in adolescents are still unclear and require further research. It also suggests that there is a non-linear relationship between DII and obesity in adolescents.

However, the precise biological mechanisms underlying the relationship between DII and obesity are not fully elucidated. Previous studies established a clear link between diet and inflammation [39–41]. Insulin resistance plays a significant role in the development of obesity. Inflammation can disrupt insulin signaling pathways, resulting in insulin resistance [42]. It is hypothesized that the inflammatory components of a high DII score may contribute to insulin resistance, thereby promoting weight gain and obesity. The composition and diversity of the gut microbiota have been implicated in obesity [43]. A pro-inflammatory diet may cause changes in the composition of gut bacteria, leading to an imbalance between beneficial and harmful bacteria. This imbalance, known as dysbiosis, can affect energy metabolism, inflammation, and fat storage, potentially contributing to weight gain and obesity [44]. Inflammatory factors can also influence the function of adipose tissue, including the secretion of adipokines and lipid metabolism [45]. A pro-inflammatory diet may disrupt the normal functioning of adipose tissue, leading to increased fat accumulation and obesity. Additionally, dietary patterns are also important risk factors leading to obesity.

The exact mechanism through which diet induces inflammation remains incompletely understood. Inflammation is significantly influenced by one’s dietary choices. While certain food items have the potential to trigger inflammation, others may aid in its reduction. The consumption of processed foods, refined carbs, sugary drinks, and unhealthy fats can contribute to the development of chronic inflammation. These particular food choices are capable of activating the immune system, thereby initiating an inflammatory response [46]. Conversely, a diet abundant in whole foods such as fruits, vegetables, whole grains, lean proteins, and healthy fats can effectively mitigate inflammation. Scientific investigations have demonstrated that adherence to the Mediterranean diet, as well as diets incorporating ample amounts of fruits, vegetables, and omega-3 fatty acids, correlates with diminished inflammation levels [47]. Implementing a low-inflammation or anti-inflammatory diet may serve as a valuable therapeutic approach in addressing obesity and its associated complications.

Inflammation is associated with various diseases, including obesity and metabolic syndromes [48, 49]. Despite the implementation of numerous measures to combat obesity, its prevalence in children and adolescents remains high which represents a major challenge for society. Therefore, it is essential to understand the relationship between DII and obesity in children and adolescents to develop dietary interventions for effectively tackling this issue. Based on our results, it appears that lowering DII in the diet may be an effective means of mitigating obesity risk, particularly in adolescents. Inflammation is a significant factor in the development of various health issues associated with obesity, such as metabolic syndrome, cardiovascular disease, and cancer (50–51). Managing dietary inflammation is critical for preventing health problems in obese children and adolescents.

The current study had several significant strengths. First, it utilized a nationally representative sample of children and adolescents in the United States, which enabled a broader reach to the general population. This sample size also allowed comprehensive anthropometric measurements and demographic data to be collected, thereby increasing the validity of the results. Moreover, collecting NHANES data on all days of each year reduced the likelihood of errors resulting from day-specific information bias, further increasing the robustness of the data and the outcome validity [52]. Additionally, employing a thorough dietary recall to determine the DII score ensured the accuracy and comprehensiveness of the dietary inflammatory potential assessment. Ultimately, this study presents valuable insights into the relationship between DII and obesity in children and adolescents, offering support for initiatives and policies that aim to mitigate childhood obesity.

The limitation of the study was the application of imputation methods to infer missing food parameters. A sensitivity analysis conducted on the imputed values yielded findings similar to those of the main analysis. Additionally, there was a lack of relevant data on Tanner stage and metabolic profile among our study participants. Despite these limitations, this study provides important insights into the relationship between DII scores and obesity in children and adolescents. However, further research is needed to confirm our findings. Additionally, future studies should consider including a more comprehensive dietary assessment tool to accurately capture the habitual diets of individuals. Furthermore, it should accumulate all relevant food parameters required to calculate the DII score. However, the DII calculated using the same number of food parameters as our study was evaluated in the NHANES, with C-reactive protein as the outcome [53]. Furthermore, because our study was cross-sectional, causality could not be directly inferred. Hence, future studies with larger sample sizes and multiple dietary assessments are required to provide further insights into the underlying mechanisms and validate our findings.

## Conclusions

Our findings indicate a possible link between DII scores and overweight/obesity, obesity, and central obesity in adolescents. These results support the hypothesis that inflammation triggered by dietary habits may play a role in the development of obesity in adolescents. Nonetheless, additional investigations are necessary to verify and expand our findings, as well as to probe the underlying biological mechanisms. Overall, these findings emphasize the critical role of a nutritious diet in the prevention and control of obesity in adolescents.

## Data Availability

The original contributions presented in the study are included in the article, further inquiries can be directed to the corresponding author.

## Abbreviations

WC: Waist circumference
WtHR: Waist-to-height ratio
NHANES: National Health and Nutrition Examination Survey
DII: Dietary Inflammation Index
MEC: Mobile examination center
BMI: Body mass index
